# National Veterinary Medical Association

**Published:** 1886-01

**Authors:** Charles A. Meyer

**Affiliations:** Rec. Sec.


### Proceedings Veterinary Medical Societies.

### NATIONAL VETERINARY MEDICAL ASSOCIATION.

The third annual meeting of this Association was held at Washington, December, 15, 16, 17. Dr. L. V. Plageman, M.R.C.V.S., called the meeting to order. T. B. Cotton, V.S., filled the place of secretary, and Dr. Charles A. Meyer, recording secretary. Dr. Hamill, D.V.S., acted as sergeant-at-arms.

Drs. H, E. Earle, M.D.V.S., Richmond Co., N. Y.; Peter Peters, V.S., New York College Veterinary Surgeons; John H. Dancer, D.V.S., Oranget N. J.; C. B. Robinson, D.V.S., Wheeling, West Va. State Board of Health; James T. Bushman, M.R.C.V.S., Washington; Thos. L. Armstrong, V.S., Indianapolis; Robt. Ward, F.R.C.V.S., Baltimore State Board of Health; Wm. H. Dougherty, V.S., Baltimore; and John A. McLaughlin, D.V.S., Providence, were present.

Letters of regret were received from the following gentlemen:—Prof. Smith, V.S., Toronto; Wm. Shepard, V.S., Ottawa, Ill.; R. Jennings, V.S., Pittsburg, Pa.; J. F. Suttcliff, Port Jervis, N.Y.; Noah Cressy, M.D.V.S., New Haven, Ct.; John Lindsay, D.V.S., Huntington, L. I.; and Chas. B. Uibel, V.S., Lancaster, Ohio.

Dr. H. E. Earle, D.V.S., introduced the discussion on Humane Associations, and the efforts of Henry Bergh, of New York, in that direction were generally commended.

There was an animated discussion on the subject of vivisection, and the viv isection of animals was to a certain extent favored, with the reservation that those to be experimented upon should not be subjected to pain or torture. All the members favored the use of anæsthetics.

Various questions relating to contagious diseases were discussed, and all the delegates favored action by the association tending to secure better protection of live stock from diseases of a contagious type, and urged the advisability of appointing qualified veterinary surgeons to official positions. It was stated that stock-raisers and the general public are not fully aware of the extent of diseases of a contagious nature that are transmittable from one animal to another, and from animals to mankind, and *vice versa*.

It was stated that great numbers of valuable stock have been lost through lack of proper treatment, and the opinion was expressed that the appointing powers should confer with the National Association, and the various State organizations of which it is composed, as to the best means of securing better protection of live stock from contagious diseases.

Dr. Plageman read a paper on the diseased condition of the nervous system called "Hydrophobia." This term he said was a misnomer, the proper name being "Rabies." The paper advanced the theory of spontaneous generation. The compound hydrogen and nitrogen, commonly known as water of ammonia, was the only safe agent to employ in the case of wounds made by rabid dogs or animals. This water of ammonia being alkaline in reaction has the power of destroying the virus.

Dr. Hamill stated that hydrophobia was, in his opinion, a blood disease and not a nervous one; that it was a contamination of the blood acting on the nerve centres. There are also various diseases which resemble, in many of their symptoms, rabies.

The transmissibility of diseases was discussed by Drs. Meyer, Dancer, Peters, Earle and Hamill, and it was declared that the facts would in time demonstrate that vaccination for many diseases would be a preventive, or at least render them milder in type. Dr. Cotton stated that vaccination for splenic fever is now claimed by a practitioner in Canada as a preventive of that disease, and he had demonstrated the usefulness of his discovery before M. Pasteur had made his researches public.

Dr. Ward, Dr. Lyman, Dr. William Dougherty and W. H. Martinet, all of Baltimore, spoke on veterinary progress and the duties its practitioners owe to their patrons, and expressed themselves pleased to see that the several States were well represented by delegates who besides being veterinarians, were graduates of regular medical colleges, showing that there is a gradual movement toward uniting the two professions.

The last morning session was on a series of questions from the Humane Society of Pa., as follows:

PENNSYLVANIA SOCIETY FOR THE PREVENTION OF CRUELTY TO ANIMALS.

PHILADELPHIA, December 16, 1885.

DEAR SIR:—This Society is now engaged in endeavoring to arrive at the most humane method of slaughtering cattle, and with that end in view, I have been requested to ask your consideration and that of your Convention to the following queries:—

1st.—Does the Israelitish method of killing—"the quick cut of gullet and windpipe"—produce a total loss of sensation?

2d.—Does a total loss of sensation result from a blow upon the head of a bullock, with hammer or axe, given with sufficient force to knock the animal down?

3d.—What is the most humane method of slaughtering cattle?

4th.—Is a bullock so sensitive as to suffer terror or agony in witnessing the death blows to and sufferings of his companions?

If, in the important proceedings of your Convention, time can be found to consider these questions, your opinions and discussions of the subjects will tend largely to the proper solution of a question which covers a wide range of inquiry. Very respectfully,

M. V. B. DAVIS, *Sec*

*To the President of the Veterinary Convention, Washington, D. C.*

The Convention decided in answer to—

*Question No.* 1—There is not a total loss of sensation.

*Question No.* 2—When properly delivered, and with sufficient force on the proper spot, then there is a total loss of sensation.

*Question No.* 3—The best method known to this body is known as the French method, which consists of a covering for the eyes with a block attached to it and on the forehead through which passes a bolt of sufficient length which is driven into the brain by a forcible blow with a mallet. then cutting the throat when felled, and bleeding immediately.

*Question No.* 4 – Animals are sensitive to the sufferings of others.

Dr. Charles A. Meyer, President of the N. Y. State Academy of Veterinary and Medical Pathology, read some interesting notes on Castration.

The emasculation of animals is one of the most common operations which the veterinarian is called upon to perform, and the custom has become almost universal, and in this country almost every male horse may be said to be a gelding, excepting those which are kept for covering, and these are mainly imported for the purpose.

Were all male horses left entire, many would be difficult to control, and life would be endangered. It is therefore a benefit to mankind that horses used for pleasure and servitude should be gelded, although the gelding no longer possesses that grace, action, form and carriage, which is so characteristic of the stallion.

By the operation of emasculation he degenerates into a comparatively mild and tractable animal, becomes reduced in stamina and constitution, and as a consequence more liable to disease; but we gain by making him a useful servant.

A brief history of castration may be of interest. The Egyptians knew the methods of gelding in a crude manner, and the Israelites and Greeks learned it from them. Cautery and ligature were the methods used. Aristotle mentions the mode of compression, whereby the testes of young animals were destroyed, but in older ones they were removed by excission. Aspyrtus remarks that in suckling colts the testes were destroyed by bruising between two blocks of wood or stones, and in older animals excission with a red-heated knife. Mago, a Carthagenian, applied split reeds to the cord, and gradually removed the testes by torsion, but in older animals applied the clamps below the epididymus, and removed the gland by excission. The method of scraping the cord, and in this way removing the gland, was practiced by the Indians, and it is claimed that through this channel it was introduced into England. The first knowledge we have of the clamps is described by Asinari, an Italian, although his ideas may have been derived from those of Mago, mentioned above.

It is not for me to discuss the different methods, as they all have but one object in view, to restrain the temper of the horse, and make him a docile servant. A few interesting observations and statistics of sequelæ, due to castration, will be mentioned.

Bouley's observations on forty-five entire horses, found that the testes of twenty-four were more developed on the left, sixteen on the right, and five equal in size. The smallest weighed two ounces, and the largest ten and one-half ounces. Hering operated on seventy-eight entire horses, aged from two and one-half to twenty-four years, and found that forty-four were larger on the left, twenty-six on the right, and eight equal in size, the smallest weighed one ounce three drachms, and the largest twelve ounces and two drachms; average weight of left testicle, seven ounces, two drachms, twenty-one grains; average weight of right testicle, seven ounces, one drachm, seventeen grains: differential average being in favor of the left, which is sixty-four grains. The following are a few statistics of sequelæ due to the operation: W. S. Adams' observations at a Remount Depot, where 10,305 animals were operated upon: 240 died, due to the operation; 116 from peritonitis, 76 gangrene, 22 hæmorrhage, 16 tetanus, 10 hernia. All were operated on by the scraping method. Peritonitis generally occurred from the third to the sixth day. Gangrene always produced a lingering death, and tetanus occurred between the fourth and twenty-seventh day. In 1856 a series of experiments were conducted by Messrs. Thacker, Shaw and Richardson. The ligature, actual cautery, caustic clamps, and the scraping methods were all tried, and resulted in favor of scraping. Lacoste,—in December, 1838, 177 horses were operated on in seven days, and with good results. On the week following, 62 were operated on, of which 42 died from peritonitis. In 1831, at a Remount Depot, 100 died, and in 1835, at Caen, 56 died from tetanus. From November 23, to December 6, 1847, of 74 horses operated upon, 32 had tetanus, of which 6 recovered. Dawson, Report of 1848,—290 were operated on by the scraping method, and no ill results. Gloay, in 1849, saw an experienced shepherd operate on 200 lambs, of which 160 died within three days.

Hurford, 1850, reported 144 operated by the scraping method, of which five died. Paton, in 1855, reported 120 mules operated by scraping method during a severe heated spell, and no ill results.

In the year 1830, a Russian, in Paris, removed the testes with his teeth, and in 1831 repeated the same performance at Viroflay. In Lapland the reindeer is delivered of his glands by old women, who gnaw through the cord with their teeth.

A professional castrator who went by the name of the "Pole," traveled to France, and located himself at Caen, where he introduced his method of castrating the horse while standing. This was in the year 1826; previous to this the torsion method was unknown in France.

This "Pole" used no means of restraint such as hobbles, ropes or twitch, but stood by the left side of the animal, near the hind leg, grasped the scrotum with the left hand, and with a knife in his right hand cut through the coverings of the testicles, seizing the protruding gland with his right hand, and with the thumb and fingers of the left he applied pressure enough to hold and gradually turned and twisted until the testicle was removed. He operated in the same manner on the other gland. Throughout the whole operation the animals remained very quiet. Mace, 1852, and Goux, 1849, operated on standing foals, applied the ligature and left the testes hanging.

The reason I mention the above facts are, that certain professionals claim the standing operation of torsion as their own.

It was first noticed in 1826, as mentioned above, the only difference being that the ecraseur is now used instead of the torsion process. The first use of the ecraseur and the gelding of horses while standing came to my notice in the year 1876. A professional castrator named C. D. House, operated in this part of the country, and in conversation with him he informed me that he had seen the operation performed in Kentucky, and there they applied the clamps on animals while standing. His success as an operator was great, and the method is now established throughout the country. The greatest castrator of these times is "Miles," of Illinois, and he has for years used the ecraseur. The scraping, the torsion, and the use of the ecraseur, are about the one and same process.

By the use of the ecraseur there is no bleeding from crushed or torn arteries, due to the complete and rapid plugging of the vessels by the turning inwards of the torn tunics, &c., and when properly used, wounds usually heal with very little local or general reaction, although under all methods sequelæ due to the operation, will happen, and with which every practitioner has to encounter, and there is none that hurts or blasts a reputation more than a case of unsuccessful castration.

It is for this reason that many veterinarians refuse to geld, especially when permanently located; they prefer the traveling professional, and treat or care for the consequences which often happen, such as pain, inflammation, hæmorrhage, peritonitis, enteritis, tetanus, gangrene, schirrus and hernia.

It is conceded by all authors and operators that many animals are predisposed to peritonitis, tetanus and sclerous cord. Climatic changes have a marked influence and many instances are recorded of these facts.

My opinion, as regards the use of the ecraseur and the standing position, is that they are far superior to other methods for the following reasons: That there is not so much liability to subjecting the animal to a nervous shock; it is done quicker, with less pain to the patient, and does not cause injury or fractures as when thrown.

The paper was ably discussed by the delegates, and a vote of thanks accorded the reader.

Dr. Thos. L. Armstrong, V.S., stated that he was called to Morgan Co., Ind., to attend on 17 mules about two-year olds, which had been operated upon with the wooden clamps. After the operation they were turned out, all crossed a stream about four feet deep, to pasture on the opposite sides, in about ten days after they showed signs of tetanus, five of which died in a few days; the remaining twelve, whose symptoms were slight, were placed in an isolated barn, and no one permitted to see them except the groom who cared for their wants. Twice a day morphine was given in the drinking water, of which they were allowed to partake as much as they choose, and strict orders to keep the place and surroundings quiet. The twelve gradually recovered.

T. B. Cotton, of Mount Vernon, Ohio, was called to see eighteen animals which had been castrated with the wooden clamps, applied by an experienced operator of twenty-four years, who claimed never to have lost a case in all those years, and that season, of the eighteen operated upon, all died.

In 1883 he saw a well known and experienced operator geld eight horses, two bitches, some chickens and ducks in one day. The first one operated on was suffering from strangles. After the first operation he operated on all the other animals. Six of the eight horses died from blood poisoning, two slowly recovered, and how the other animals fared he did not know; the ecraseur was used, and the cause was inoculation from the strangles patient as he did not clean the instrument.

C. B. Robinson, V.S., stated that Prof. McEcheron, of Montreal, Canada, advised his students never to castrate monorchids, as the practitioner might be censured, should any patient die after the operation of castration.

Dr. J. H. Dancer, D.V.S., Orange, N. J., reported sixteen horses operated on by a professional castrator, and well known throughout Pennsylvania, they were owned by a wealthy stock raiser, and nine of them died.

Dr. L. V. Plageman, M.R.C.V.S., castrated a great many, and with good results. He was called to operate on three horses, two-year-olds and owned by one gentleman, and the three died within four days. Operated with clamps and actual cautery.

Dr. H. E. Earl, of Richmond County, N. Y., always operated with the ecraseur, and has so far always had the best results.

Dr. Robt. Ward, F.R C.V.S., cited cases, and stated that there was danger in the operation, by any method. He had for years been a co-worker with Prof. Geo. Fleming, of London: advised the use of the ecraseur, used it himself, and it was advocated by all the English schools.

A motion was made and carried that the ecraseur be recommended as the most scientific instrument for castration, known to this body.

In the afternoon of the last session the delegates convened in Baltimore, Md., to listen to an elaborate discourse on "Milk," by Dr. R. Ward.

The following officers were elected for the ensuing year: President-James Hamill, New York; first vice-president, T. B. Cotton, Mount Ver, non, Ohio: second vice-president, H. E. Earle, West New Brighton, N. Y.; third vice-president, C. B. Robinson, Wheeling, W. Va.; recording secretary, T. L. Armstrong, Indianapolis, Ind.; corresponding secretary, Peter Peters, New York; treasurer, L. V. Plageman, Brooklyn, N. Y.; trustees, Charles A. Meyer, John A. Dancer and H. E. Earle.

The Convention decided to hold its next meeting in November, 1886, at some city in the State of Ohio, to be decided upon by the President.

CHARLES. A. MEYER, D.V.S., *Rec. Sec.*

